# Metagenomics of the Viral Community in Three Cattle Slurry Samples

**DOI:** 10.1128/MRA.01442-18

**Published:** 2019-02-14

**Authors:** Pavelas Sazinas, Slawomir Michniewski, Branko Rihtman, Tamsin Redgwell, Aurelija Grigonyte, Amelia Brett, Bethany Guck, Rebecca Smith, Steven P. Hooton, Jon L. Hobman, Andrew D. Millard

**Affiliations:** a School of Life Sciences, University of Warwick, Coventry, United Kingdom; b School of Biosciences, University of Nottingham, Sutton Bonington Campus, Sutton Bonington, Leicestershire, United Kingdom; c Department of Genetics and Genome Biology, University of Leicester, Leicester, United Kingdom; University of California, Riverside

## Abstract

The diversity of viruses in slurries from dairy farming remains largely uncharacterized. Here we report viral diversity found in cattle slurry from a dairy farm in the East Midlands in the United Kingdom. The same slurry tank was sampled in three consecutive years, and the viral fraction was isolated and sequenced.

## Abstract (precis)

The diversity of viruses in slurries from dairy farming remains largely uncharacterized. Here we report viral diversity found in cattle slurry from a dairy farm in the East Midlands in the United Kingdom.

## ANNOUNCEMENT

The United Kingdom has approximately 1.8 million head of dairy cattle, which produce ∼14,000 million liters of milk and millions of tons of manure per annum. The separated solid manure and liquid cow slurry that are produced are widely used as a fertilizer. Due to legislation to protect nitrate-vulnerable zones, animal slurries can be applied to land only at certain times of the year; thus large quantities of slurry are often stored. Although the bacterial fraction of the cow rumen has been characterized in some detail (1, 2) and to a lesser extent the viral fraction (3), relatively little is known about the bacterial or viral composition of slurry, the main constituent of which will be cow feces but will also include other farm wastes. The aim of this work was to begin to investigate the diversity of viruses in stored cow slurry, using metagenomics.

Samples were collected from a dairy farm slurry tank in the East Midlands in the United Kingdom, in three consecutive years from 2015 to 2017. The viral fraction was isolated based on the following method: the slurry was mixed at a ratio of 1:10 with phosphate-buffered saline (PBS), before centrifugation at 10,000 g at 4°C for 20 min to remove bacterial cells and other debris, and the supernatant was removed to sterile centrifuge tubes. Further rounds of centrifugation were carried out to remove any bacterial cells that became resuspended during removal of the supernatant. The supernatant was then sequentially filtered through 0.45-µm and 0.22-µm pore-size syringe filters (Sartorius) before concentration of the viral fraction using an Amicon 100-kDa cutoff membrane filter. Contaminating free-host DNA in the viral fraction was reduced by addition of DNase I to a final concentration of 1 U/µl at room temperature for 1 h. DNA was extracted from 1 ml of the viral fraction using a modified phenol:chloroform extraction method (4). Sequencing libraries were prepared with 1-ng input DNA with an Illumina Nextera XT DNA sample kit as per manufacturer’s protocol (Illumina, San Diego, CA). Sequencing was performed on an Illumina MiSeq instrument using the paired-end 2 × 250-bp protocol (v2), which resulted in 12.8 million reads across three samples. Reads were trimmed with Sickle v1.33 with the parameters “–l 20 -t 20” (5). Metagenomes were coassembled using MEGAHIT with the parameters “–kmin 21 –kmax 249 –kstep 10” (6). A total of 616,440 contigs were assembled, of which 62,964 were greater than 1 kb, and the largest contig had 53.06 kb. To evaluate bacterial contamination, SortMeRNA v2.1 was used to identify rRNA reads (7), having an average content of ∼0.03%, which is commensurate with other viromes and suggestive they are not heavily contaminated with bacterial DNA (8). The obtained genomic data are being used to describe the viral diversity, in particular the bacteriophages found within cow slurry.

### Data availability.

This whole-genome shotgun project has been deposited at DDBJ/EMBL/GenBank under the accession number PRJEB28736.
